# Bioactive diterpenoids impact the composition of the root-associated microbiome in maize (*Zea mays*)

**DOI:** 10.1038/s41598-020-79320-z

**Published:** 2021-01-11

**Authors:** Katherine M. Murphy, Joseph Edwards, Katherine B. Louie, Benjamin P. Bowen, Venkatesan Sundaresan, Trent R. Northen, Philipp Zerbe

**Affiliations:** 1 Department of Plant Biology, University of California-Davis, One Shields Avenue, Davis, CA USA; 2 Integrative Biology, University of Texas, Austin, 2405 Speedway, Austin, TX USA; 3 Environmental Genomics and Systems Biology, Lawrence Berkeley National Laboratory, M/S 100PFG100, 1 Cyclotron Road, Berkeley, CA 94720 USA; 4 Joint Genome Institute, Lawrence Berkeley National Laboratory, 1 Cyclotron Road, Berkeley, CA 94720 USA

**Keywords:** Secondary metabolism, Microbiome

## Abstract

Plants deploy both primary and species-specific, specialized metabolites to communicate with other organisms and adapt to environmental challenges, including interactions with soil-dwelling microbial communities. However, the role of specialized metabolites in modulating plant-microbiome interactions often remains elusive. In this study, we report that maize (*Zea mays*) diterpenoid metabolites with known antifungal bioactivities also influence rhizosphere bacterial communities. Metabolite profiling showed that dolabralexins, antibiotic diterpenoids that are highly abundant in roots of some maize varieties, can be exuded from the roots. Comparative 16S rRNA gene sequencing determined the bacterial community composition of the maize mutant *Zman2* (*anther ear 2*), which is deficient in dolabralexins and closely related bioactive kauralexin diterpenoids. The *Zman2* rhizosphere microbiome differed significantly from the wild-type sibling with the most significant changes observed for Alphaproteobacteria of the order Sphingomonadales. Metabolomics analyses support that these differences are attributed to the diterpenoid deficiency of the *Zman2* mutant, rather than other large-scale metabolome alterations. Together, these findings support physiological functions of maize diterpenoids beyond known chemical defenses, including the assembly of the rhizosphere microbiome.

## Introduction

Extensive research in recent years has demonstrated the composition and importance of rhizosphere microbial communities to plant health and fitness^1–3^. The cooperative partnership of microbes and plants has been attributed largely to an exudation of photosynthate sugars from the plant in exchange for nutrient supply and protection against biotic and abiotic stress, ultimately contributing to increased plant vigor and yield^4,5^. Despite extensive characterization of species- and tissue-specific microbial communities and how these vary in their ecological and genetic contexts, our understanding of the mechanisms by which plants recruit and maintain root-associated microbial communities is still limited^5–11^.

As the most economically important crop in the United States, maize (*Zea mays*) has been the subject of long-standing research to improve crop yield and stress resilience traits^12^. The “core” microbiome of maize has provided insights into the presence of specific phyla in and near maize roots^6,13,14^. A study of 27 maize inbred lines across developmental stages and geographical locations demonstrated maize genotype as a replicable factor in defining the microbiome, with significant variation due to the different genetic backgrounds among inbred lines. Five “core” Operational Taxonomic Units (OTUs), all in the phyla Proteobacteria, were found to be present in all samples^14^. Drawing on these detailed insights into the maize microbiome, further research has ventured into determining the chemical signaling factors that determine microbiome composition and variation across genotypes. Specialized metabolites mediate various plant interactions with the environment and other organisms, and several major bioactive metabolite groups have been identified in maize, including diterpenoids, sesquiterpenoids, oxylipins, and benzoxazinoids^15–20^. Given their distinct structures, bioactivities and abundance across different maize tissues and developmental stages, maize specialized metabolites can be hypothesized to play a critical role in the plant communication with root-borne microbes and thus help determine the root microbiome. Recently, benzoxazinoids were shown to influence the maize response to stress via altered rhizosphere microbial communities mediated by 6-methoxy-benzoxazolin-2-one (MBOA)^21^. Additional benzoxazinoid mutant studies showed changes to fungal and bacterial communities as a result of benzoxazinoid deficiency^22^. Furthermore, benzoxazinoid-deficient mutants were shown to feature substantially altered root metabolomes, suggesting alterations in the microbiome may not be solely attributed to a lack of specific benzoxazinoids, but rather global changes in root metabolites in mutant plants^23^. Across different studies, there further is a large degree of variation in the observed differences in alpha and beta diversity in benzoxazinoid mutants, largely dependent on developmental stage, environmental conditions, and mutational and genetic background^21–23^.

**Figure 1:**
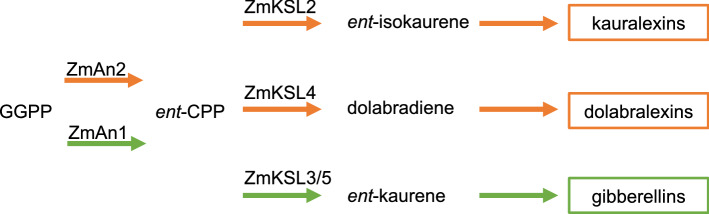
Schematic overview of key enzymes involved in diterpenoid biosynthesis in maize. Abbreviations: GGPP, (*E*,*E*,*E*)-geranyl geranyl diphosphate; *ent*-CPP, copalyl diphosphate; An, anther ear; KSL, kaurene synthase-like. Orange arrows represent pathways en route to specialized, defensive metabolites. Green pathways represent gibberellin hormone biosynthesis.

The diverse group of terpenoid metabolites also has shown to be critical in mediating above- and below‐ground interactions between plants and other organisms, including microbes^24^. Kauralexins and dolabralexins are two major diterpenoid groups in maize that have demonstrated or predicted roles in biotic and abiotic stress responses^16–18,25^. Kauralexins show stress-elicited accumulation in several tissues, including stems and scutellum, and mediate quantitative defenses against fungal pathogens such as species of *Fusarium*, *Aspergillus* and *Cochliobolus*, as well as insect pests including the European corn borer (*Ostrinia nubilalis*)^17,26–28^. The more recently discovered group of dolabralexins shows pathogen-inducible accumulation predominantly in roots and, like kauralexins, have strong growth-inhibitory activity against *Fusarium* pathogens^18^. In addition to their defensive potential against biotic stressors, both kauralexin and dolabralexin production was shown to increase in response to abiotic stress, such as drought or oxidative stress^16,18^. Kauralexins and dolabralexins derive from a common precursor, *ent*-copalyl pyrophosphate (*ent*-CPP), which is also shared with the gibberellin (GA) biosynthetic pathway critical for plant growth (Fig. 1)^17,29^. Two catalytically redundant diterpene synthase (diTPS) enzymes, ANTHER EAR 1 (ZmAn1) and ANTHER EAR 2 (ZmAn2) control *ent*-CPP formation in maize^29,30^. Genetic studies revealed that ZmAN1 is critical for GA biosynthesis, whereas ZmAN2 feeds *ent*-CPP into kauralexin and dolabralexin biosynthesis, thus enabling a pathway partition separating precursor flux toward primary and secondary (i.e. specialized) diterpenoid pathways^29–31^ (Fig. 1). This pathway separation is supported by the phenotype of the *Zman2* mutant, which features a loss of function in the *Zman2* gene through a stable Ds insertion from the Activator (Ac) and Dissociation (Ds) system^16,29^. *Zman2* has kauralexin and dolabralexin deficiency but normal GA levels^16^. *Zman2* has a normal growth phenotype, but is more susceptible to biotic and abiotic stress than its wild type (WT) sibling, which is consistent with the protective bioactivity of kauralexins and dolabralexins^16^ and suggests a possible role of these metabolites in broader plant–microbe interactions, including root microbiota. To test this hypothesis, we combined microbial 16S rRNA sequencing and metabolite profiling of the diterpenoid-deficient *Zman2* mutant compared to its WT sibling to investigate the role of diterpenoids in shaping the root microbial communities.

## Results

### Dolabralexins are secreted from of maize roots

**Figure 2:**
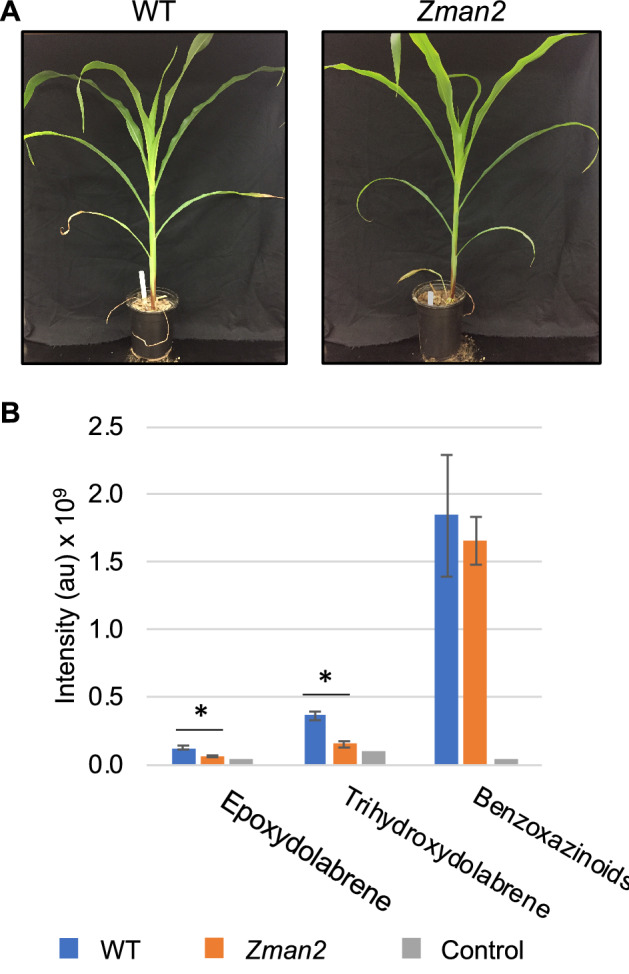
Phenotype and metabolite abundance in maize *Zman2* mutants and WT plants. (**A**) Representative images of *Zman2* mutant plants and the corresponding WT sibling used in this study. (**B**) Average intensity of metabolites in maize root exudate samples using positive mode LC–MS/MS analysis. Peak area given for epoxydolabrene [M + H], trihydroxydolabrene [M + H-H2O], and benzoxazinoids, with the last based on detection of metabolites containing BOA (1,3-benzoxazol-2-one (BOA) [M + H]). * represents significant difference between two sample types, p ≤ 0.05. Two-tailed t-tests were used for benzoxazinoids; one-tailed t-tests were used for dolabralexins, since they are predicted to be enriched in WT and deficient in *Zman2.* Error bars represent standard error. n = 5 (WT, *Zman2*); Control represents one extraction of nutrient water, and demonstrates LC–MS/MS background.

To examine a possible role of maize diterpenoids in plant-microbiome interactions, we utilized the *Zman2* mutant genotype in comparison to its isogenic WT sibling^16,29^. Previous studies showed that 30-day-old WT maize plants significantly accumulate dolabralexins, and to a lesser extent kauralexins, in roots^16^, whereas the *Zman2* mutant genotype is almost completely devoid of these diterpenoids^16–18^. Despite the deficiency of both kauralexins and dolabralexins in *Zman2* root tissue, mutant plants did not show an apparent phenotype under well-watered conditions (Fig. 2A), consistent with prior reports describing largely unaltered root and shoot weight, developmental features, and GA and zealexin levels in the *Zman2* mutant^16^. *Zman2* plants were used as a control for analyzing root metabolite exudation due to the deficiency of diterpenoids, and subsequent analysis of the microbiome and metabolome.

To determine a possible ability of maize diterpenoids to affect the rhizosphere microbiome, we first tested if diterpenoids can be exuded from maize roots. For this purpose, 38-day-old *Zman2* and WT maize plants were grown on soil, then gently cleaned and suspended for 48 h in nutrient water. After removing the plants, metabolites were extracted from the nutrient water using an equal volume of ethyl acetate and analyzed by LC–MS/MS against authentic metabolite standards. As a positive control, the benzoxazinoid 1,3-benzoxazol-2-one (BOA), known to be secreted from maize roots, was measured and used as a standard to detect BOA and other benzoxazinoids metabolites with similar mass spectra. Benzoxazinoids were found to be present in both *Zman2* and WT plant exudates, while absent in nutrient water without plants, as expected (Fig. 2B). Of the known dolabralexins for which standards were available—epoxydolabrene and trihydroxydolabrene—only trace amounts were detected in nutrient water after incubation of the *Zman2* mutant (Fig. 2B), as expected based on the known mutant phenotype^18^. Trihydroxydolabrene and epoxydolabrene were both significantly enriched in the WT root exudate samples than *Zman2* or nutrient water without plants (Fig. 2B). Epoxydolabranol, the third dolabralexin for which a standard was available, was not detected in mutant nor WT plant exudates.

### Maize root microbial communities are distinct by compartment

The *Zman2* mutant genotype and its corresponding WT sibling serve as a tool to investigate the effect of diterpenoids, or the lack thereof, on the maize root microbial community^16,18^. Based on the growth conditions of previous research on the mutant genotype, showing an enrichment of dolabralexins and to a lesser extent kauralexins, we used one-month-old *Zman2* and isogenic WT sibling plants to comparatively examine the impact of diterpenoid-deficiency on the maize root microbiome. Representative plant images are shown in Fig. 2A.

Microbiomes of the rhizosphere (1–2 mm of soil outside the root) and endosphere (inside the root), the latter representing root samples after removal of rhizosphere and rhizoplane microbes through washing and sonication of the roots, were analyzed. Bulk soil without plants was used as a control to examine background soil microbial communities. The 16S rRNA gene (V4 region) was sequenced using Illumina MiSeq and sequences were clustered into operational taxonomic units (OTUs) using the QIIME pipeline and the Greengenes database^32^. After filtering to remove mitochondrial and chloroplast OTUs, 4,259 distinct OTUs remained. Following standard protocols for analyzing data with a binomial distribution, such as the MiSeq data generated in this study, OTU counts were normalized by relative abundance, in which the counts were taken as a percentage of the total number of OTU counts for a sample^33–35^. This method was used rather than rarefaction methods, which are at risk for discarding so as not to discard low abundance OTUs^36^. The DESeq2 package in R, which is used to analyze data with a binomial distribution such as the data generated in this study, was used to determine which OTUs were enriched or depleted in the WT and *Zman2* samples^37^.

**Figure 3:**
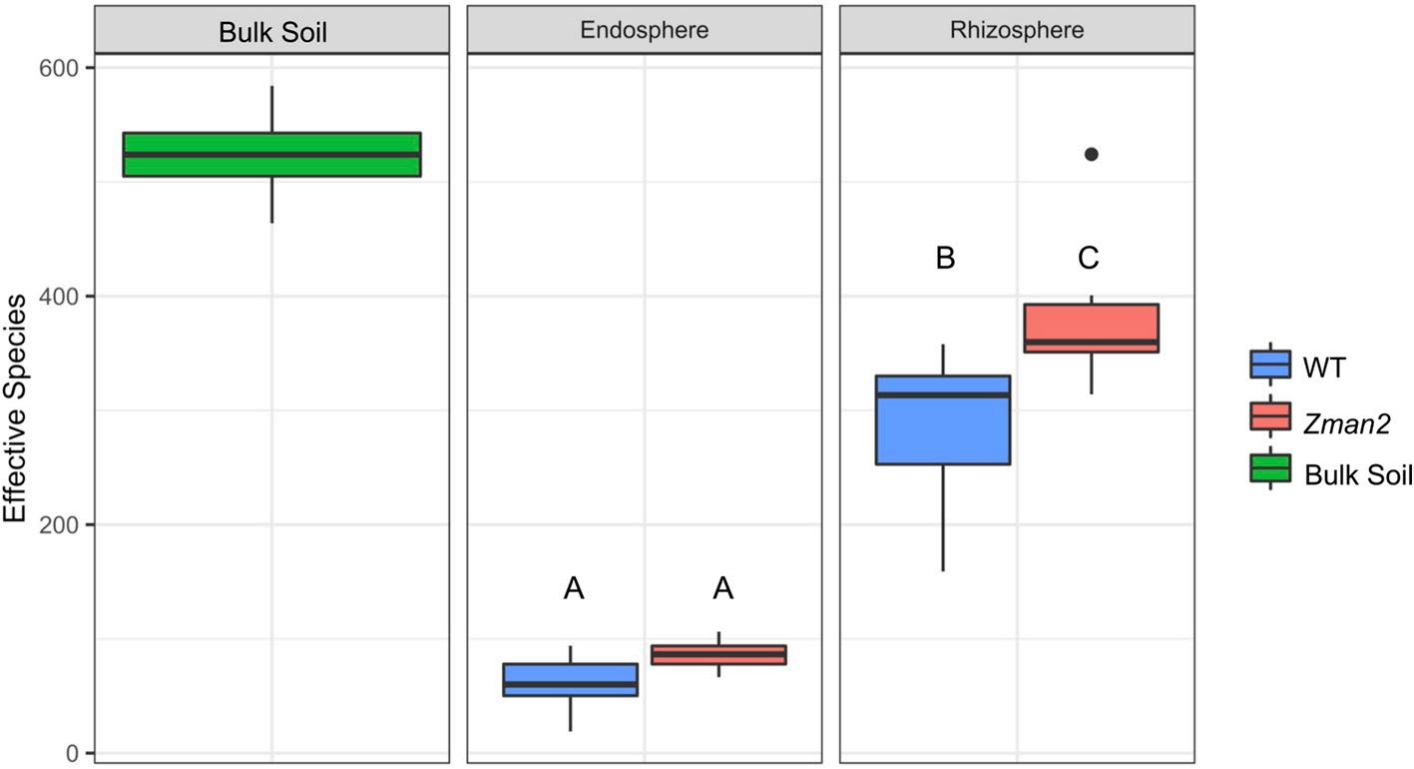
Alpha diversity of each sample type, as measured by the Shannon’s H index. Letters represent significantly different measurements, p ≤ 0.05. All statistics are available in Supplemental Table 2. n = 5 (bulk soil) or n = 6 (all plant samples).

**Figure 4:**
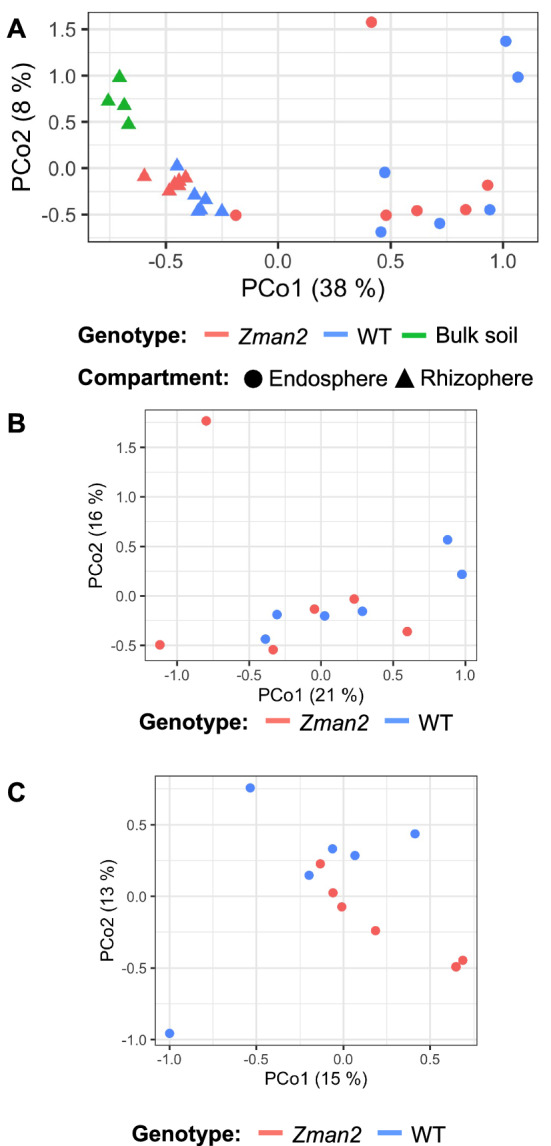
Principal Coordinate Analysis (PCoA) using Bray distances for (**A**) all samples, (**B**) endosphere only, and (**C**) the rhizosphere only. Each point represents an individual plant or soil sample. Percentage in the axis labels represents the eigenvalue, or percent of variation explained by that axis. n = 5 (bulk soil) or n = 6 (all plant samples).

Consistent with previous research in maize and other plant species^7,38^, the microbial communities of the two plant compartments and the bulk soil were all statistically distinct. The alpha diversity, as measured by the Shannon index, showed the greatest diversity of microbes in bulk soil, with reduced diversity in the rhizosphere and further reduction in the endosphere (Fig. 3, statistics reported in Supplemental Table 2). Sequencing depth was used as a covariate to account for variance due to experimental variables and not any possible variance due to sequencing depth. A permutational multivariate analysis of variance (PERMANOVA) was used to measure the diversity between samples (beta diversity) and showed that, when accounting for all factors, compartment accounts for 31% of the variation between samples (p < 0.001) (Supplemental Table 1). This was confirmed by a principle coordinate analysis (PCoA), in which compartment was the greatest source of variation (Fig. 4A).

**Figure 5:**
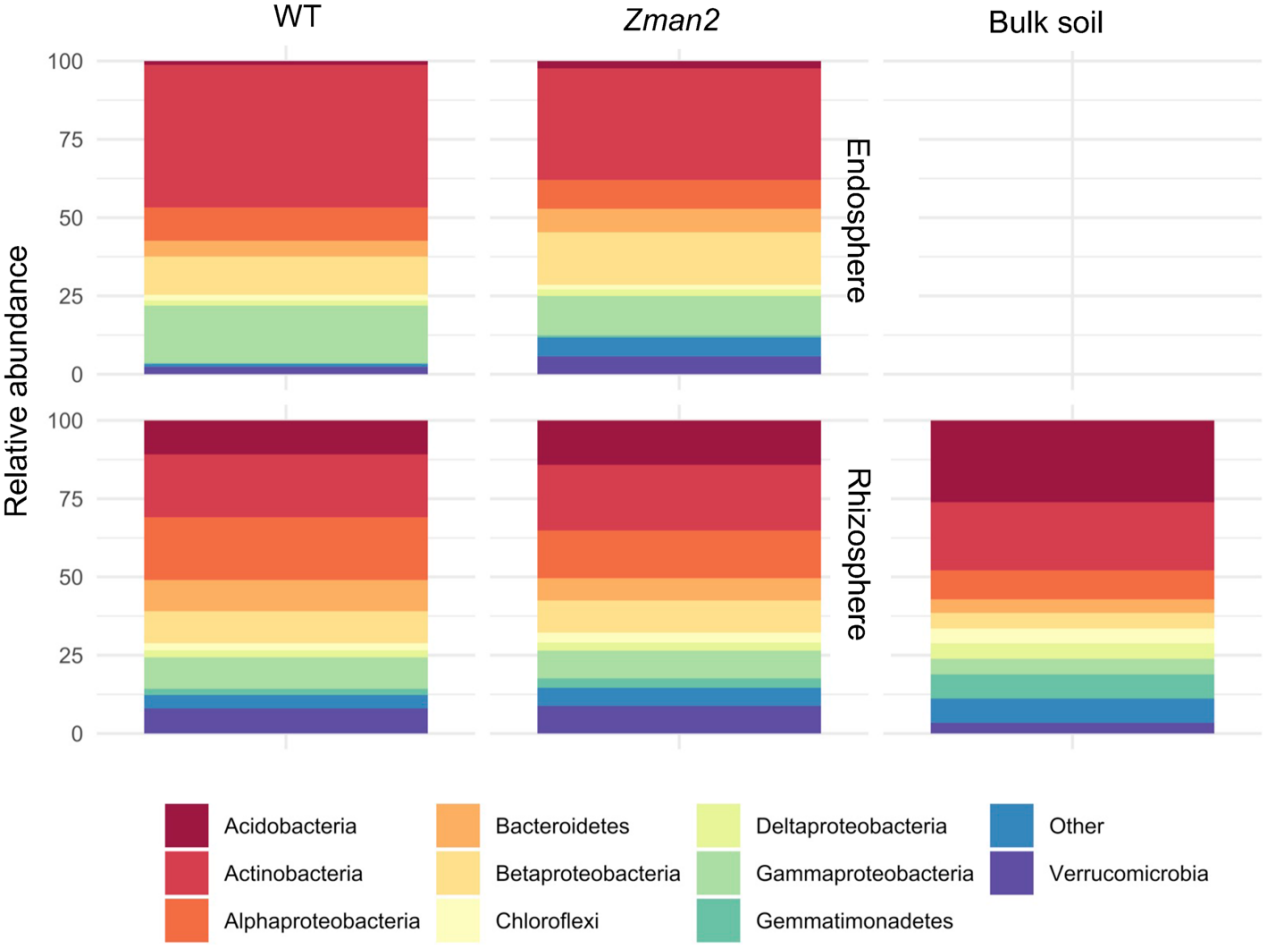
Distribution of the ten most abundant phyla for each sample type. Bars represent the relative abundance of all OTUs within each of the top 10 most abundant phyla. n = 5 (bulk soil) or n = 6 (all plant samples).

A total of 547 OTUs, in 19 phyla (out of 34 total phyla represented by all data points), were enriched in the rhizosphere as compared to the endosphere, whereas 63 OTUs in 8 phyla were enriched in the endosphere as compared to the rhizosphere, as determined using the DESeq2 package in R^37^. Among the 10 most abundant phyla plotted for each sample type, some phyla were found to be enriched in both compartments, whereas the rhizosphere was predominantly enriched for OTUs in the phyla Actinobacteria, Acidobacteria, and Alphaproteobacteria (Fig. 5).

The endosphere did not demonstrate significant differences in beta diversity or alpha diversity attributed to genotype, as determined by PERMANOVA and PCoA or Shannon index, respectively (Fig. 4B). No individual OTUs were significantly enriched or depleted in regards to genotype in the endosphere as analyzed by generalized linear models using the DESeq2 package in R^37^. Because there was no apparent difference, all further analysis focuses on the rhizosphere compartment only, and the bulk soil and endosphere samples were omitted from further analyses.

### The *Zman2* mutant features a distinct microbial community composition

Next, the impact of genotype on the rhizosphere microbiome composition was assessed. Significant differences in the microbiome composition were observed between WT and *Zman2* plants, with genotype accounting for 10.7% of the variation in the rhizosphere (p < 0.05) (Supplemental Table 1). *Zman2* plants harbor a more diverse microbiome as determined by a greater alpha diversity compared to the WT sibling in the rhizosphere (Fig. 3, Supplemental Table 2). Six OTUs were more abundant in WT plants, whereas none were enriched in *Zman2* samples in the rhizosphere (Supplemental Fig. 1; OTU abundances by sample type plotted in Supplemental Fig. 2). Of the six OTUs, all were assigned to Alphaproteobacteria belonging to the order Sphingomonadales, three of which were assigned to the genus Sphingobium, whereas the remaining OTUs were unclassified at the genus level.

### Wild type and mutant plants have largely indistinguishable metabolomes

**Figure 6:**
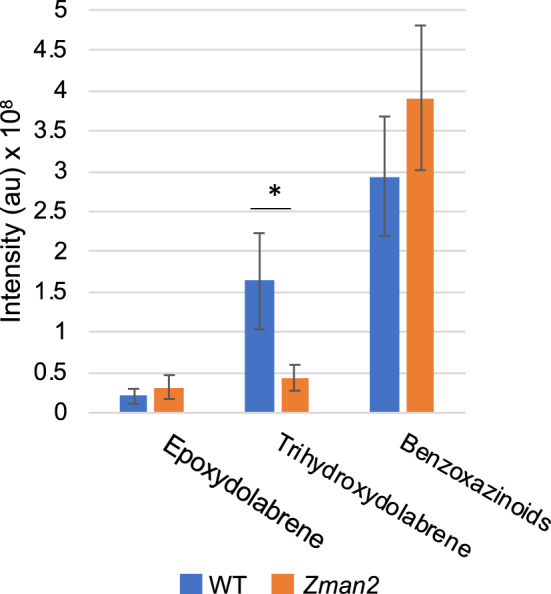
Average intensity of metabolites in maize root samples using positive mode LC–MS/MS peak area. Peak area based on epoxydolabrene [M + H], trihydroxydolabrene [M + H-H2O], and benzoxazinoids, with the last based on detection of metabolites containing BOA (1,3-benzoxazol-2-one (BOA) [M + H]). * represents significant difference between two samples p ≤ 0.1. Two-tailed t-tests were used for benzoxazinoids; one-tailed t-tests were used for the remaining metabolites, since they are predicted to be enriched in WT and deficient in *Zman2*; n = 4 (*Zman2*) or n = 5 (WT). Error bars represent standard error.

To verify that differences in microbiome composition can be attributed to a deficiency in diterpenoids in the roots of *Zman2* plants, metabolite profiling using both targeted and untargeted LC–MS/MS analysis was performed on the same root samples used for microbial composition analysis. Targeted metabolite analysis of the major dolabralexin metabolite, trihydroxydolabrene (THD), confirmed via a standard that THD was near absent in the *Zman2* mutant while present in WT (Fig. 6). Epoxydolabranol was not found in either mutant nor WT plants (Supplemental Fig. 3), while epoxydolabrene was found to be lowly abundant in both mutant and WT plants (Fig. 6), presumably because of their conversion to THD. Mirror plots demonstrate these identifications, as well as their absence in the mutant plants (Supplemental Fig. 3). This observation is consistent with previous research, showing low levels of dolabralexin and kauralexin metabolites in *Zman2*, predictably due to *ent*-CPP derived from ZmAn1 activity^16^. As a control, benzoxazinoid abundance was calculated using BOA as a standard, and found to be present in both *Zman2* and WT roots without significant differences in abundance. Using BOA for LC–MS/MS generates multiple peaks with similar mass spectra due to various benzoxazinoids compounds, and their total area was analyzed here (Supplemental Fig. 3).

**Figure 7:**
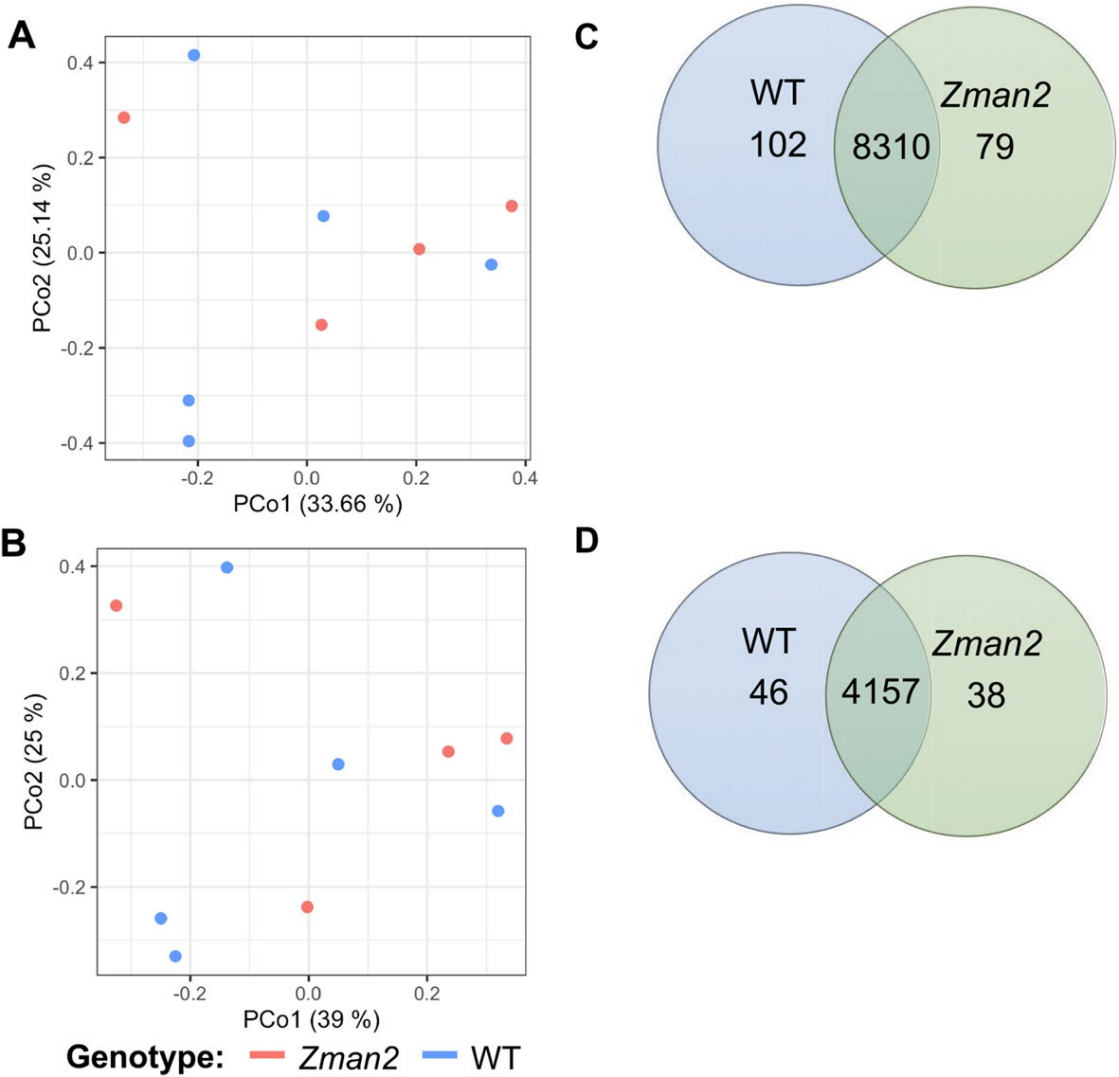
Principal Coordinate Analysis (PCoA) of the metabolome for all root samples in (**A**) positive mode or (**B**) negative mode using LC–MS/MS. Each point represents an individual plant sample. Percentage in the axis labels represents the eigenvalue, or percent of variation explained by that axis. Number of enriched metabolites detected using LC–MS/MS in positive mode (**C**) or negative mode (**D**), as measured by a generalized linear model at the level of p ≤ 0.05, for each genotype. Overlap are not significantly different; n = 4 (*Zman2*) or n = 5 (WT).

Parallel untargeted metabolomics analysis also did not indicate significant variance in the global metabolite profiles of the WT and *Zman2* plants, determined by principle component analyses (Fig. 7A,B). Furthermore, PERMANOVA analysis based on all features (dominant mass ions and corresponding specific retention times) demonstrated genotype did not significantly impact the metabolome using either positive or negative ionization modes (Supplemental Table 3). Although the PERMANOVA and PCoA demonstrated no significant difference overall between genotypes, a generalized linear model was used to identify individual metabolites that may be significantly enriched or depleted. Performing analyses in both positive and negative ionization modes, a total of 102 and 46 metabolites were enriched in WT as compared to 79 and 38 enriched in *Zman2,* respectably by ionization mode (Fig. 7C,D, Supplemental Table 4). Annotation of the remaining metabolites with distinct abundance in WT and *Zman2* roots by comparison to mass spectral databases identified significant matches for 16 compounds (Supplemental Table 4). Consistent with the targeted metabolite profiling, THD was identified among the 10 most abundant compounds in the 102 enriched metabolites in WT roots (ID positive-2360). The remaining metabolites could not be annotated with high confidence, but probably represent so far uncharacterized dolabralexin- or kauralexin-derived metabolites (Supplemental Fig. 4, Supplemental Table 4). The metabolite group containing THD (ID positive-2360), represented as connected nodes in the metabolic network generated in Cytoscape, is connected to two other features that were not significantly enriched or depleted and unannotated, but may represent dolabralexin-type molecules given the similarity of their mass spectra. An additional compound enriched in WT was annotated as mesterolone, a triterpenoid, yet features mass spectra highly similar to that of epoxydolabrene (Supplemental Table 4). The remaining metabolites were at least one order of magnitude less abundant as compared to THD in WT plants (Supplemental Fig. 4, Supplemental Table 4). Although the linear models show some metabolites enriched or depleted in WT versus *Zman2* plants (Fig. 7C,D), the global metabolome is not significantly altered and THD represents one of the most significantly different metabolites in its abundance.

## Discussion

The dynamic interrelations between plants and their species-specific root microbiota directly influence plant health and stress tolerance^4^. Despite the importance of these mutualistic relationships, the complex chemical mechanisms coordinating inter-organismal interactions remain largely elusive. In particular, limited knowledge exists on how specific metabolites, blends thereof, and the corresponding pathways impact plant–microbe interactions and microbial community assembly. For example, recent maize studies illustrated that mutant genotypes deficient in benzoxazinoid metabolites (specifically MBOA) showed an altered stress response mediated by the influence of MBOA on the below-ground microbial community, thus underscoring the importance of these metabolite-guided plant–microbe interactions on plant health^21–23^. The microbiome and metabolome analyses performed in this study support the hypothesis that specific groups of bioactive diterpenoids in maize, namely dolabralexins and/or kauralexins, contribute to the assembly of the rhizosphere microbiome.

Although the underlying secretion mechanisms require further study, presence of dolabralexins in maize root exudates supports a role of these compounds in below-ground plant–microbe interactions (Fig. 2). Microbiome analysis of the root microbial communities showed no significant influence of genotype on endosphere communities using distance-based methods (Fig. 4B). It appears plausible that diterpenoids do not impact endophytic microbes due to the spatial separation of endophytic microbes that predominantly colonize the apoplast^39,40^, whereas functionalized diterpenoids accumulate intracellularly as demonstrated in several plant species, and are exuded into the rhizosphere as shown here^41–43^. Our results showing distinct rhizosphere microbial communities of *Zman2* and its WT sibling with a more diverse root microbiome alpha diversity in *Zman2* (Fig. 3), provide evidence supporting a role of diterpenoids in the microbiome assembly by reducing the community diversity. This difference in diversity supports the hypothesis that dolabralexins directly inhibit the growth and/or propagation of specific rhizobia bacteria. This is further supported by the distinct beta diversity between *Zman2* and WT (Fig. 4C), with genotype accounting for 10.7% of the variation between samples. Notably, the significant differences between the two genotypes were defined by only a few OTUs, most of which were assigned to the order Sphingomonadales (Supplemental Figs. 1 and 2). Sphingomonads have been reported to degrade phenolic compounds and utilize them as carbon sources^44^, and were among the OTUs displaying the greatest heritable variation (H^2^) across maize lines of the NAM (Nested Association Mapping) diversity panel^14^. Considering the variation of dolabralexins across selected maize inbred lines^18^, it can be speculated that not only phenolics, but also maize-specific diterpenoids mediate the interaction with species of Sphingomonadales.

The overall increased microbiome diversity in the *Zman2* mutant differs from previous research supporting that a greater microbial diversity promotes crop resistance to soil pathogens (Fig. 3)^45,46^, given that previous work has shown *Zman2* to be more susceptible to fungal pathogens^29^. Considering these findings in association with the demonstrated anti-microbial activity of both kauralexins and dolabralexins^17,18^, the relationship between the disease-preventative properties of dolabralexins and bacterial diversity remains more complex, and the influence of dolabralexins may not be directly on the bacterial communities, but possibly indirect via fungal communities and other microbe-microbe interactions, in addition to or instead of direct impacts on growth of rhizobia bacteria.

Recent research provided insight into the effect of benzoxazinoids on the microbiome and their importance to plant health. Contrasting the significant role of diterpenoids in determining alpha diversity shown in this study (Fig. 3), benzoxazinoids did not change alpha diversity, but impacted bacterial beta diversity and specific phyla and OTUs to varying degrees depending on the experimental conditions and genotypes used^21–23^. Thus, it appears plausible that the impact of plant age, soil type, environmental stimuli, genetic background, and/or sampling on metabolite-microbiome interactions contribute to these contrasting results. The level of variation in beta diversity are comparable to the 10.7% of beta diversity explained by diterpenoids in this study. Moreover, the difference in the impact of benzoxazinoids and diterpenoids on the rhizosphere alpha diversity and community composition point toward distinct functionalities of different metabolite classes in maize-microbiome interactions. Recent analysis of two rice mutants, *Oscps2* and *Oscps4*, deficient in rice-specific diterpenoids showed indistinguishable rhizosphere microbiomes from their WT siblings, suggesting that maize diterpenoids are distinct in their ability to modify the rhizosphere microbiome^47^.

The lack of changes in the global metabolome of WT and *Zman2* roots is consistent with WT phenotype of *Zman2* mutant plants under healthy conditions (Fig. 7)^16^, and supports a major role of dolabralexin and/or kauralexin bioactivity, rather than other large metabolic perturbations caused by the ZmAN2 loss of function, on the observed microbiome alterations. While the overall metabolomes were not significantly different, PERMANOVA and PCoA studies identified a number of unidentified metabolites with distinct abundances in WT and *Zman2* plants. Here were select metabolites not yet identified as dolabralexins or kauralexins that were altered in their abundance between the mutant and WT. These could be not-yet-identified dolabralexins or kauralexins products, breakdown products of these metabolites, or other fluctuations. Interestingly, several of these significantly enriched or depleted compounds featured mass fragmentation patterns suggesting that they represent yet unknown dolabralexin- and kauralexin-type compounds with possible functions in the plants interaction with the rhizosphere microbiome. Notably, recent research investigating the root metabolome and microbiome in maize mutants deficient in the biosynthesis of selected benzoxazinoid compounds found a different scenario, where mutant plants displayed significant metabolic changes across many pathways in PCoA and other statistical methods^23^. These results underscore the importance of metabolite analysis to understand the broader metabolic implications of pathway mutations especially within complex, branching metabolite networks, so as to not attribute microbiome changes to a single absent metabolite, but possibly due to larger changes throughout the metabolic network in the mutant plants.

Using a defined pathway mutant, the present study supports a role of species-specific bioactive maize diterpenoids in shaping the rhizosphere microbiome diversity and composition. These findings expand our insight into diterpenoid functions in Poaceous crops beyond well-established anti-microbial and anti-feedant bioactivities. Such deeper knowledge of the mechanisms underlying natural plant–microbe interactions will be critical for ultimately enabling broader agricultural applications.

## Methods

### Root exudate analysis

Seeds of *Zman2* and its isogenic wild type sibling (both in the W22 background) were obtained from Dr. Eric Schmelz (UC San Diego). Seeds (n = 6 for each genotype) were planted and grown in potting soil (Sunshine Mix #1, Sungro Horticulture, 75–85% Canadian Sphagnum peat moss, 25–15% perlite) in 1 gallon pots in a greenhouse and watered with nutrient water, containing calcium nitrate (0.6 g/L water), Growmore 4–13-38 (0.3 g/L water), and magnesium sulfate (0.3 g/L water). Growmore contains the following nutrients by weight: 4% total nitrogen, 18% phosphoric acid, 38% soluble potash, 0.5% magnesium, 0.2% boron, 0.1% copper, 0.4% iron, 0.2% manganese, 0.01% molybdenum, and 0.1% zinc.

After 38 days, plants were removed from pots and the roots were gently washed with deionized water so as not to cause tissue damage. The nutrient solution pH was 3.36, determined using an Ohaus Starter2000 pH meter. The plants were then placed in 2.8 L Erlenmeyer flasks with the nutrient water previously described and suspended with tape such that only the roots were in the nutrient water. Flasks were wrapped in aluminum foil to prevent light stress to the roots and placed in a growth chamber (conditions as detailed below). After 48 h, plants were removed, and the nutrient water was filtered through a metal strainer to remove any possible tissue debris. Metabolites were extracted from the exudate water by adding 700 mL of ethyl acetate to 700 mL exudate water and leaving at 4 °C for 24 h. The organic solvent layer was then separated and concentrated using a rotary evaporator for metabolite analysis. Nutrient water containing no plants was used as a control.

### Plant growth conditions

Soil was collected at a UC Davis research field site (coordinates 38.531152, -121.783182) by collecting approximately 6 inches of top soil using bleached shovels and collecting the soil in sterile bags. The field site had grown maize for one year, and at the time of collection (November 19, 2016) was fallow and had the stover previously turned under after the summer harvest. The soil at this field site is a silty clay loam, as determined using the standard “texture by feel” method for soil classification^48^. The soil was then mixed in the sterile bags by using the sterile shovel to stir and mix until the soil appeared homogenous. The soil was then distributed to 2.37 L pots that were sterilized using 3% bleach wash. The soil was not sieved, and any large rocks or soil chunks were removed by hand during pot filling.

Plants were grown in a growth chamber in the pots in order to control for all other environmental conditions. The growth chamber was set to a 16/8 h day/night cycle, with a 26/22 °C day/night temperature cycle. Seeds of *Zman2* and WT plants were sterilized in 3% (v/v) bleach for one hour, then washed five times with deionized water, and planted approximately 3 cm deep in the pots with maize field soil. Pots were distributed in the growth chamber in a block design to mitigate location effects. *Zman2* and WT plant microbiomes and corresponding metabolomes were measured for six biological replicates each using bulk soil (no plants) as a control. Pots were watered every other day with 175 mL of nutrient water (see contents in root secretion assay methods), and tissue was harvested on the 45^th^ day as described below.

### Microbiome sample collection

Sample collection and processing was adapted from Edwards et al.^7^. In brief, plants were carefully removed from the soil, and gently shaken until ~ 2 mm of soil adhering to the root remained. n = 5 (bulk soil) or n = 6 (all plant samples). The roots were then transferred to a 50 mL falcon tube contained sterile phosphate buffer saline (PBS, 137 mM NaCl, 2.7 mM KCl, 10 mM Na_2_HPO_4_, 1.8 mM KH_2_PO_4_) and placed on ice. For analysis of the rhizosphere microbiome, these roots in the PBS in falcon tubes on ice were shaken using sterile forceps to remove the soil from the root surface and soil samples were stored at 4 °C until further processing the next day. Gentle shaking with the forceps and careful observation ensured that no roots were broken and in the rhizosphere sample. For analysis of the endosphere microbiome, the above root samples were placed into fresh PBS buffer in a new 50 mL falcon tube and sonicated three 3 times for 10 s each, followed by placing the roots in fresh PBS buffer again to remove any rhizoplane microbes. Using these roots, ~ 4 cm sections of the primary root (beginning 2 cm below the root-shoot junction) was cut, placed in a new tube, frozen in liquid N_2_, and stored at − 80 °C until further processing. Bulk soil samples from soil 2 cm below the surface were collected using a sterile scoop and stored in PBS buffer at 4 °C until sample processing the next day.

### DNA extraction

All DNA was extracted using the MoBio PowerSoil DNA extraction kit and eluted in 50 µL of DEPC-treated water. The rhizosphere samples were concentrated by pipetting 1 mL of the rhizosphere soil in PBS into a 2 mL tube and centrifuged for 30 s at 10,000×*g*. The supernatant was discarded and the soil was used for DNA extraction. The endosphere samples were homogenized and ground in liquid N_2_ for DNA extraction with the MoBio Powersoil DNA kit.

### 16S rRNA gene amplification, quantitation, and sequencing

The V4 region (515 to 806 bp of the 16S rRNA gene) of the 16S rRNA gene was amplified according to Edwards et al.^49^. In brief, PCR was performed using Qiagen HotStart HiFidelity polymerase with the following parameters for each mix: 6.25 µL water, 2.5 µL buffer, 1.25 µL of 10 µM forward primer, 1.25 µL of 10 µM reverse primer, 0.25 µL HotStart polymerase, and 1 µL of DNA. Specific primer pairs, containing unique 12 bp barcode adaptors on each end of the forward and reverse primers were used for each reaction. Samples without DNA were used as negative controls. A touchdown PCR program was used with the following parameters: 95 °C for 5 min; 7 cycles of 95 °C for 45 s, 65 °C for 1 min decreasing at 2 °C per cycle, and 72 °C for 90 s; 30 cycles of 95 °C for 45 s, 50 °C for 30 s, 72 °C for 90 s; a final extension of 72 °C for 5 min; and samples were held at 4 °C. Only samples producing single amplicon bands as verified by agarose gel electrophoresis were considered for further analysis, and all samples were maintained for downstream analysis.

Amplicons were purified to remove primers using AmPure XP beads (Beckman Coulter). Here, beads were added to each PCR reaction, incubated at room temperature for 5 min, and placed on a magnet for 2 min to separate the beads. After removal of the supernatant, the beads were washed with 70% ethanol twice. The ethanol was then allowed to evaporate and the beads were resuspended in 50 µL water, mixed well, and placed again on a magnet to remove the supernatant containing the desired PCR products. DNA concentrations were measured using a Qubit, and pooled to reach samples of equimolar concentrations. The pooled samples were cleaned as described above, separated by agarose gel electrophoresis and the 400 bp amplicons were extracted using a NucleoSpin Gel and PCR Clean-up kit (Macherey–Nagel). Libraries were made and sequencing was performed at the UC Davis Genome Center using 250 × 250 paired end, dual index Illumina MiSeq sequencing.

### Sequence analysis

Sequences were analyzed as previously described by Edwards et al.^7^. In brief, sequences were demultiplexed based on individual barcodes using a custom R script, and assembled into single sequences using Pandaseq. Sequences were then clustered into OTUs with the NINJA-OPS pipeline using 97% pairwise sequence identity referenced against the Greengenes 16S rRNA sequence database (version 13_8)^32^.

In total, 1,802,959 high-quality sequences were obtained with a median read count of 31,204 per sample, and a range of 1699–87,795 (All data is available in Sequence Read Archive, Sequence Read Archive repository, BioProject ID PRJNA600272 [https://www.ncbi.nlm.nih.gov/sra/PRJNA600272]). Using the QIIME pipeline, reads were clustered based on 97% sequence identity into operational taxonomic units (OTUs) and were annotated using QIIME and the Greengenes database^32^, resulting in 7181 microbial OTUs. Chloroplast and mitochondrial OTUs represented 65 OTUs and were removed, along with low-abundance OTUs (less than 5% of the total sample), leaving 4258 total OTUs. OTU counts were then normalized by relative abundance, which was used rather than rarefaction methods so as not to discard low abundance OTUs^36^.

All statistical analysis of the OTU table generated by QIIME^32^ were analyzed using custom R scripts (version 3.6.1)^50^. Alpha-diversity was measured using the “Shannon” method in the R package vegan^51^. Principle coordinate analysis (PCoA) were conducted using unconstrained principles and Bray distances in the R package vegan^51^. PERMANOVA (permutational multivariate analyses of variance) analysis was performed using the R package vegan function adonis to measure beta-diversity^51^. The DESeq2 package^52^ was used to identify OTUs and phyla whose abundance was differentially affected by our experimental variables. Phyla counts were derived by aggregating raw counts for OTUs at the phylum level within each sample. After analysis by DESeq2, the results were compiled and tidied using the biobroom package^53^. Plots were visualized using the ggplot2 in the tidyverse package^54^. All scripts generated in this study have been deposited to GitHub (https://github.com/kmurphy61/maizemicrobiome).

### Metabolite extraction

The remaining roots (~ 1 g fresh weight) used for endosphere microbiome analysis (see microbiome sample collection) were homogenized and ground in liquid nitrogen. Because of availability of tissue, the number of plant samples were reduced for metabolite extraction as compared to microbiome DNA extraction; n = 4 for *Zman2*, n = 5 for WT. Samples were then placed in a 2 mL glass vial and metabolites extracted by incubation in 2 mL methanol overnight at 4 °C with gentle rocking. Samples were centrifuged for 10 min at 4000 × g and the methanol phase transferred to a new vial using a glass pipette, air-dried, and resuspended in 100 µL methanol.

### Metabolite analysis

For metabolite analysis by liquid chromatography tandem mass spectrometry (LC–MS/MS), samples were spiked with 4 µM internal standard mixture of deuterium-labeled lipids (Cat# 110899, 857463P, 861809O, 110922, 110922, 110921, 110918, 110579, 110544, Avanti Polar Lipids, Inc) and 1 µg/mL ABMBA (2-Amino-3-bromo-5-methylbenzoic acid, Sigma). UHPLC reverse phase chromatography was performed using an Agilent 1290 LC coupled with a QExactive Orbitrap MS (QE = 139) (Thermo Scientific, San Jose, CA). Chromatography was performed using a C18 column (Agilent ZORBAX Eclipse Plus C18, Rapid Resolution HD, 2.1 × 50 mm, 1.8 µm) at a flow rate of 0.4 mL/min and injection volume varied from 0.9 to 3.5 µL to normalize against sample dry weight. Samples were run on the C18 column at 60 ºC equilibrated with 100% buffer A (100% LC–MS water w/ 0.1% formic acid) for 1 min, following by a linear dilution of buffer A down to 0% with buffer B (100% acetonitrile w/ 0.1% formic acid) over 7 min, and followed by isocratic elution in 100% buffer B for 1.5 min. Full MS spectra were collected ranging from *m*/*z* 80–2,000 at 60,000 to 70,000 resolution in both positive and negative mode, with MS/MS fragmentation data acquisition using an average of stepped 10–20-40 and 20–50-60 eV collision energies at 17,500 resolution. For targeted analysis, product identification by comparison to standards was performed where authentic standards were available.

For untargeted analysis, exact mass and retention time coupled with MS/MS fragmentation spectra were used to identify compounds. Features—high intensity signals narrowly contained at a given retention time and *m*/*z*—were detected using the MZMine software v 2.24 (http://dx.doi.org/10.1093/bioinformatics/btk039). Data was filtered to remove MS/MS fragment ions within ± 17 Da of the precursor m/z, and subsequently filtered to remove all but the top six ions in the ± 50 Da throughout the spectrum. Precursor and fragment ion tolerance was 0.05 Da. Features that showed a significantly different abundance (peak height) using generalized linear models, calculated using custom R scripts and the lm() function^50^, with statistical analysis results in Supplemental Table 3 and significantly encriched or depleted features listed in Supplementary Table 4. Generalized linear models are linear regression models used to determine if a particular feature is significantly different in abundance between two genotypes. All features were annotated using Global Natural Products Social Molecular Networking (GNPS)^55–59^. In short, a Feature-Based Molecular Networking workflow was used to assign features to a molecular network with a cosine score above 0.7 and more than six matched peaks. The maximum size of a molecular family was 100, and low scoring edges were removed to meet this threshold. The spectra were then searched against GNPS spectral libraries and annotated with the top hit, if there was one. Complete annotations, features present, and Cytoscape visualization networks are available online (https://gnps.ucsd.edu/ProteoSAFe/status.jsp?task=a748e519752249e2a912dd3d466db98d). All scripts are available on GitHub (https://github.com/kmurphy61/maizemicrobiome.git). Lists of significantly different features enriched or depleted in each sample type are available in Supplemental Table 4.

## Supplementary Information

Supplementary Legends.

Supplementary Tables.

Supplementary Tables.

Supplementary Figures.

## Data Availability

The datasets generated during and/or analyzed during this study are available in the Sequence Read Archive repository, BioProject ID PRJNA600272 [https://www.ncbi.nlm.nih.gov/sra/PRJNA600272]. Complete metabolite annotations and Cytoscape visualization networks are available (https://gnps.ucsd.edu/ProteoSAFe/status.jsp?task=a748e519752249e2a912dd3d466db98d). The code used to analyze these datasets are available in the GitHub repository, https://github.com/kmurphy61/maizemicrobiome.git.
