# Supplementary material for: Bioactive diterpenoids impact the composition of the root-associated microbiome in maize (Zea mays)
Source: Sci Rep. 2021 Jan 11;11:333. doi: 10.1038/s41598-020-79320-z (PMC7801432; doi:10.1038/s41598-020-79320-z)
Supplement: Supplementary file 1 — Supplementary Legends. [file 41598_2020_79320_MOESM1_ESM.docx]

**Supplemental Fig. 1**: Differentially expressed OTUs in the rhizosphere, as measured by a linear model for genotype using the DESeq2 package in R. Overlap is non-significantly different OTUs, at the level of p **≤** 0.05. n = 6 for each genotype.

**Supplemental Fig. 2**: Relative abundance of OTUs found to be significantly enriched using linear models by the DESeq2 package in R; n= 6 for each genotype.

**Supplemental Fig. 3:** Mirror plots depicting representative LC/MS chromatograms and fragmentation spectra from WT root extract, *Zman2* root extract, and a purified standard. BOA was used as a basis to detect other benxozazinoid metabolites containing BOA as a fragment ion (m/z 136), such as DIMBOA-Glc, whose mass spectra is consistent with that in WT, and thus peak area was summarized as “benzoxazinoids.”

**Supplemental Fig. 4**: Feature intensity of features found to be enriched or depleted in WT vs. *Zman2* in positive and negative ionization modes of LC/MS-MS; n = 5 for WT, n = 4 for *Zman2*.

**Supplemental Table 1:** Results from statistical analysis for analysis of the beta diversity of all samples using PERMANOVA using relative abundance normalization. n= 5 (bulk soil) or n = 6 (all plant samples).

**Supplemental Table 2:** Results from the statistical analysis of the alpha diversity of all samples using the Shannon’s H index and relative abundance normalization. n= 5 (bulk soil) or n = 6 (all plant samples).

**Supplemental Table 3:** Results from the statistical analysis of the metabolomes of WT vs. *Zman2* roots using PERMANOVA n = 4 (*Zman2*) or n = 5 (WT).

**Supplemental Table 4:** Significantly different metabolite features - high intensity signals narrowly contained at a given retention time and *m*/*z* - in LC-MS/MS enriched in WT or *Zman2* roots and their predicted identities; n = 4 (*Zman2*) or n = 5 (WT).
