# Supplementary material for: Bioactive diterpenoids impact the composition of the root-associated microbiome in maize (Zea mays)
Source: Sci Rep. 2021 Jan 11;11:333. doi: 10.1038/s41598-020-79320-z (PMC7801432; doi:10.1038/s41598-020-79320-z)
Supplement: Supplementary file 2 — Supplementary Tables. [file 41598_2020_79320_MOESM2_ESM.pdf]

Supplementary Table 1

Compartment	Term	Degrees of freedom	Sum of Squares	Mean of Squares	F value	R^2	p. value
Using relative abundance normalization and all sample types							
NA	Compartment	1	1.05279	1.05279	9.761	0.3085	0.001
NA	Genotype	1	0.12044	0.12044	1.116	0.0352	0.264
NA	Compartment: Genotype	1	0.08206	0.08206	0.761	0.0240	0.646
NA	Residuals	20	2.15712	0.10785	NA	0.6321	NA
NA	Total	23	3.41242	NA	NA	1.0000	NA
Using relative abundance normalization, no bulk soil samples, and analyzing each genotype separately							
Rhizosphere	Genotype	1	0.05826	0.05826	1.1952	0.3085	0.044
Rhizosphere	Residuals	10	0.48743	0.48743	NA	0.0352	NA
Rhizosphere	Total	11	0.54569	NA	NA	1.0000	NA
Endosphere	Genotype	1	0.14425	0.14425	0.8639	0.6321	0.727
Endosphere	Residuals	10	1.66969	1.66969	NA	1.0000	NA
Endosphere	Total	11	1.81394	NA	NA	1.0000	NA

Supplementary Table 2

Compartment	Term	Degrees of freedom	Sum of Squares	Mean of Squares	F value	p. value
Using relative abundance normalization and all sample types.						
NA	Genotype	1	23493	23493	7.915	0.0107
NA	Compartment	1	410953	410953	138.462	1.92x10^-10
NA	Genotype:Compartment	1	8233	8233	2.774	0.1114
NA	Residuals	20	59360	2968	NA	NA
Using relative abundance normalization, no bulk soil samples, and analyzing each genotype separately						
Rhizosphere	Genotype	1	29770	29770	5.429	0.0421
Rhizosphere	Residuals	10	54837	5484	NA	NA
Endosphere	Genotype	1	1956	1955.6	4.324	0.0643
Endosphere	Residuals	10	4523	452.3	NA	NA

Supplementary Table 3

Term	Degrees of freedom	Sum of Squares	Mean of Squares	F value	R^2	p. value
Positive Mode						
Genotype	1	0.00509	0.00509	1.1046	0.1363	0.39
Residuals	7	0.03229	0.00460	NA	0.8637	NA
Total	8	0.03731	NA	NA	1.0000	NA
Negative Mode						
Genotype	1	0.00385	0.00385	0.9413	0.1185	0.46
Residuals	7	0.02862	0.00409	NA	0.8815	NA
Total	8	0.03247	NA	NA	1.0000	NA
