# Supplementary material for: Bioactive diterpenoids impact the composition of the root-associated microbiome in maize (Zea mays)
Source: Sci Rep. 2021 Jan 11;11:333. doi: 10.1038/s41598-020-79320-z (PMC7801432; doi:10.1038/s41598-020-79320-z)
Supplement: Supplementary file 4 — Supplementary Figures. [file 41598_2020_79320_MOESM4_ESM.pdf]

Bioactive diterpenoids impact the composition of the root-associated microbiome in maize (Zea mays)

Katherine M. Murphy, Joseph Edwards, Katherine B. Louie, Benjamin P. Bowen, Venkatesan Sundaresan, Trent R. Northen, Philipp Zerbe

Supplementary Figures

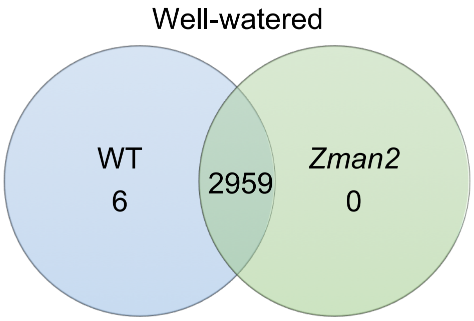

Supplemental Fig. 1: Differentially expressed OTUs in the rhizosphere, as measured by a linear model for genotype. Overlap is non-significantly different OTUs, at the level of $p \leq 0.05$. n = 6 for each genotype.

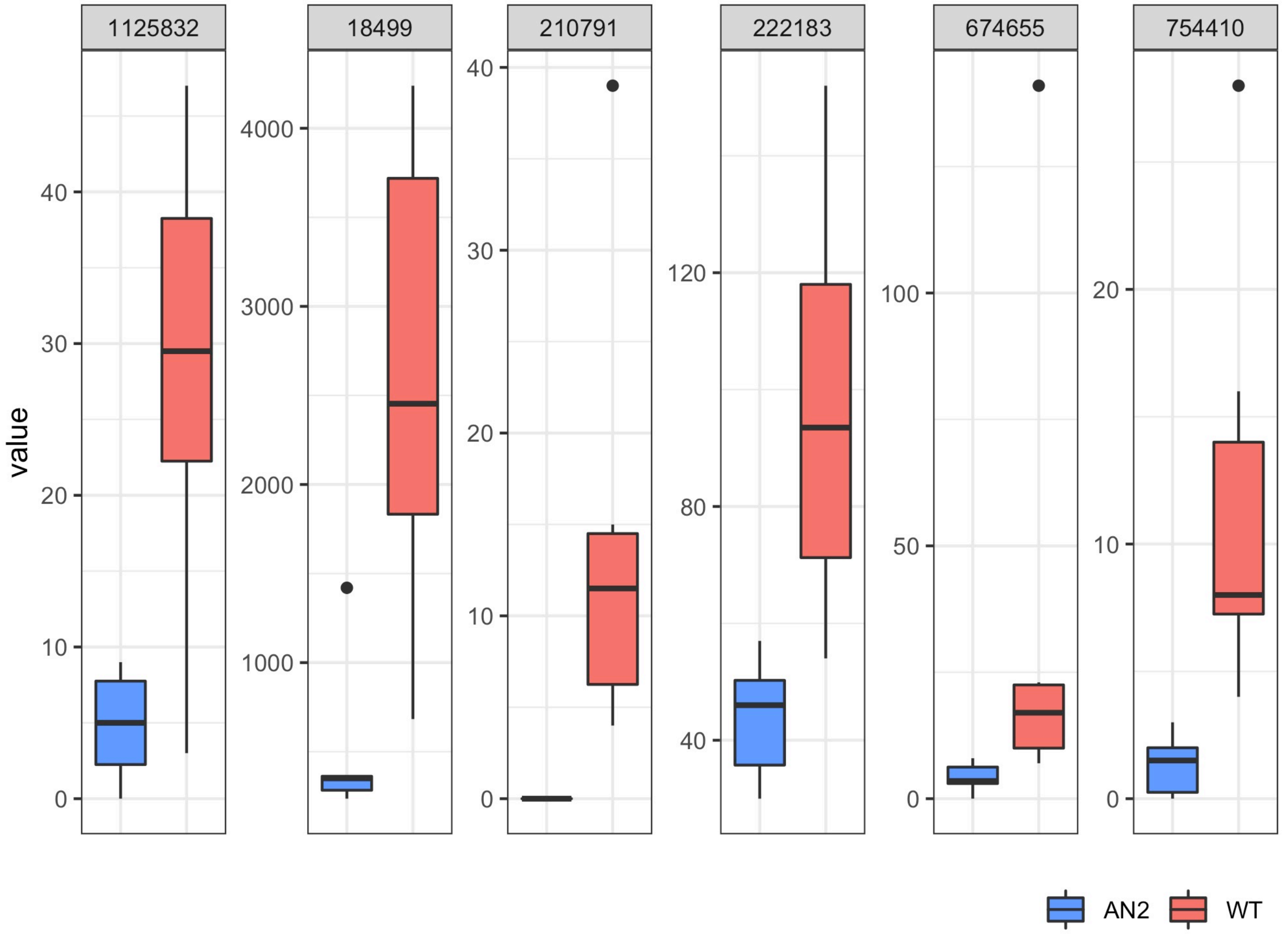

Supplemental Fig. 2: Relative abundance of OTUs found to be significantly enriched using linear models; n= 6 for each genotype.

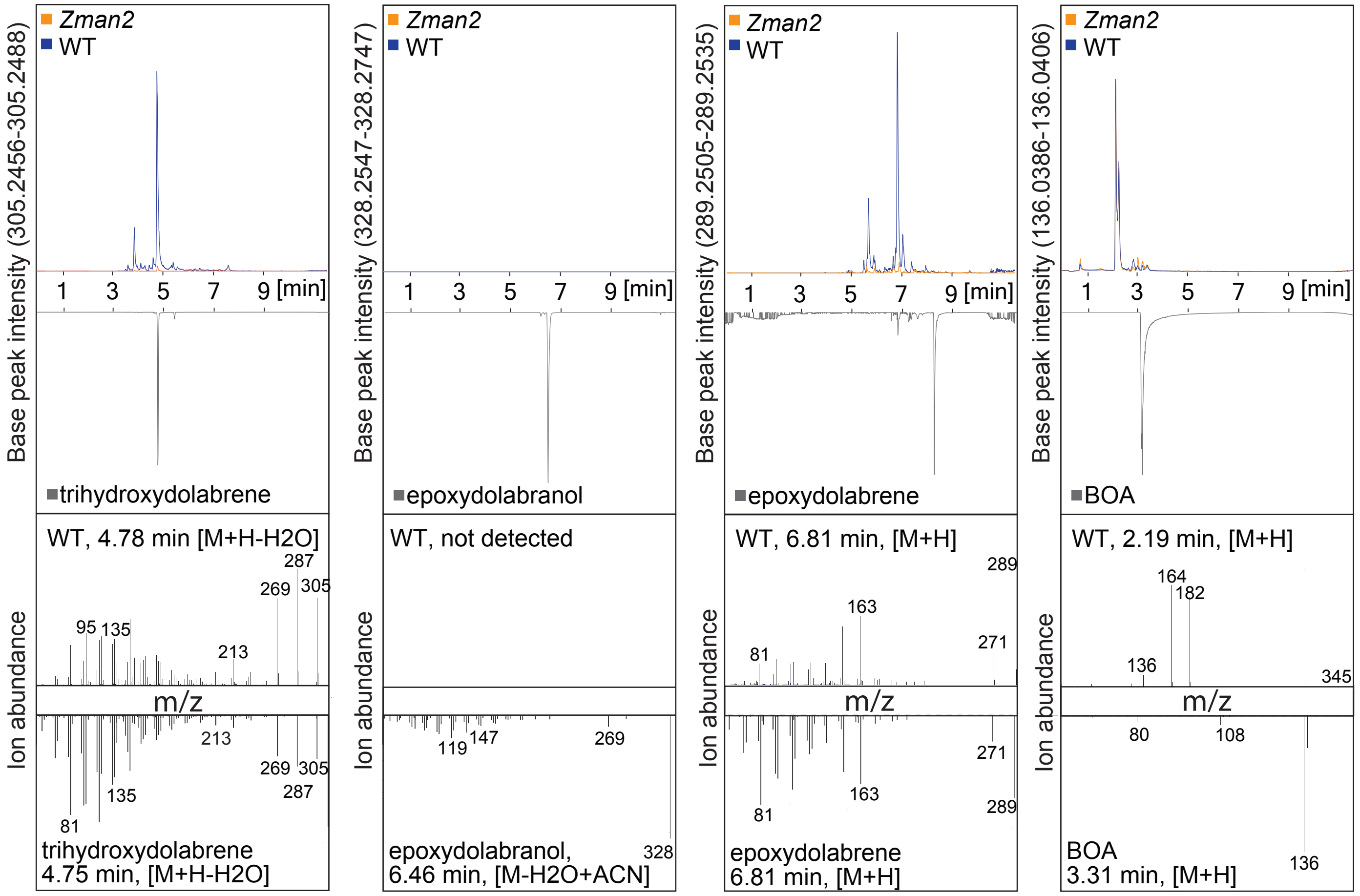

Supplemental Fig. 3: Mirror plots depicting representative LC/MS chromatograms and fragmentation spectra from WT root extract, *Zman2* root extract, and a purified standard. BOA was used as a basis to detect other benxozazinoid metabolites containing BOA as a fragment ion (m/z 136), such as DIMBOA-Glc, whose mass spectra is consistent with that in WT, and thus peak area was summarized as “benzoxazinoids.”

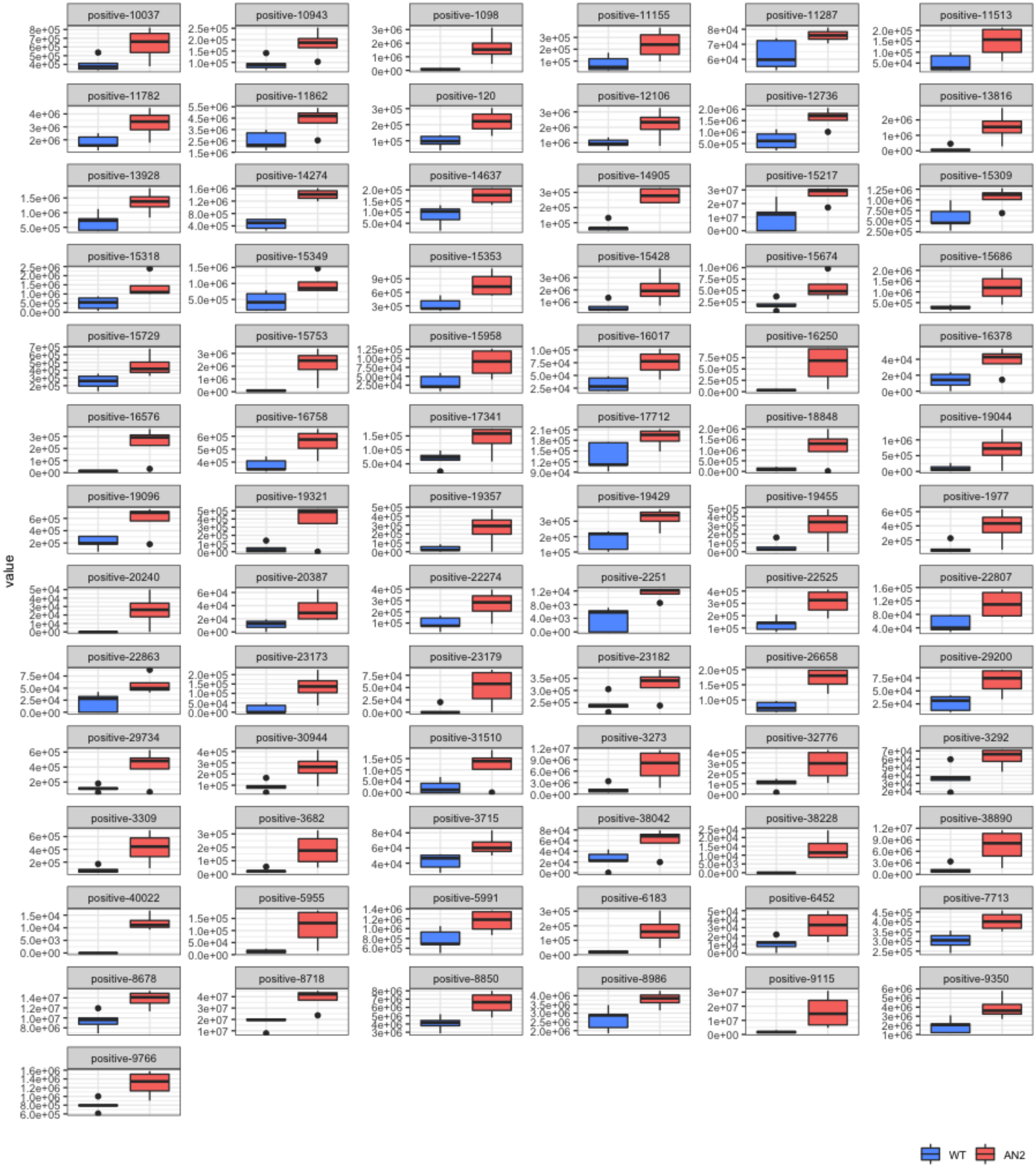
positive-10037
positive-10943
positive-1098
positive-11155
positive-11287
positive-11513
positive-11782
positive-11862
positive-120
positive-12106
positive-12736
positive-13816
positive-13928
positive-14274
positive-14637
positive-14905
positive-15217
positive-15309
positive-15318
positive-15349
positive-15353
positive-15428
positive-15674
positive-15686
positive-15729
positive-15753
positive-15958
positive-16017
positive-16250
positive-16378
positive-16576
positive-16758
positive-17341
positive-17712
positive-18848
positive-19044
positive-19096
positive-19321
positive-19357
positive-19429
positive-19455
positive-1977
positive-20240
positive-20387
positive-22274
positive-2251
positive-22525
positive-22807
positive-22863
positive-23173
positive-23179
positive-23182
positive-26658
positive-29200
positive-29734
positive-30944
positive-31510
positive-3273
positive-32776
positive-3292
positive-3309
positive-3682
positive-3715
positive-38042
positive-38228
positive-38890
positive-40022
positive-5955
positive-5991
positive-6183
positive-6452
positive-7713
positive-8678
positive-8718
positive-8850
positive-8986
positive-9115
positive-9350
positive-9766
value
WT
AN2

POsitive

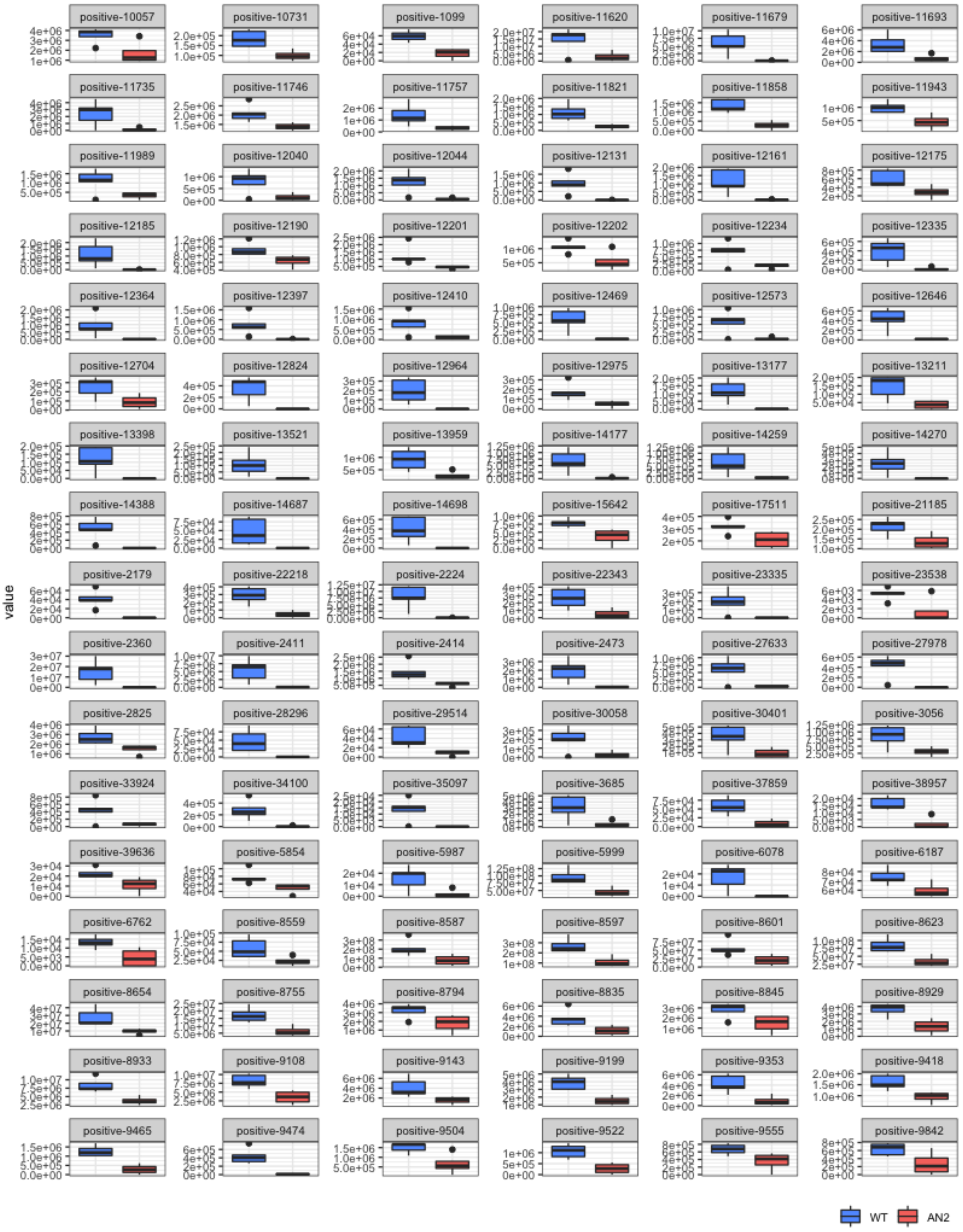
positive-10057
positive-10731
positive-1099
positive-11620
positive-11679
positive-11693
positive-11735
positive-11746
positive-11757
positive-11821
positive-11858
positive-11943
positive-11989
positive-12040
positive-12044
positive-12131
positive-12161
positive-12175
positive-12185
positive-12190
positive-12201
positive-12202
positive-12234
positive-12335
positive-12364
positive-12397
positive-12410
positive-12469
positive-12573
positive-12646
positive-12704
positive-12824
positive-12964
positive-12975
positive-13177
positive-13211
positive-13398
positive-13521
positive-13959
positive-14177
positive-14259
positive-14270
positive-14388
positive-14687
positive-14698
positive-15642
positive-17511
positive-21185
positive-2179
positive-22218
positive-2224
positive-22343
positive-23335
positive-23538
positive-2360
positive-2411
positive-2414
positive-2473
positive-27633
positive-27978
positive-2825
positive-28296
positive-29514
positive-30058
positive-30401
positive-3056
positive-33924
positive-34100
positive-35097
positive-3685
positive-37859
positive-38957
positive-39636
positive-5854
positive-5987
positive-5999
positive-6078
positive-6187
positive-6762
positive-8559
positive-8587
positive-8597
positive-8601
positive-8623
positive-8654
positive-8755
positive-8794
positive-8835
positive-8845
positive-8929
positive-8933
positive-9108
positive-9143
positive-9199
positive-9353
positive-9418
positive-9465
positive-9474
positive-9504
positive-9522
positive-9555
positive-9842
value
WT
AN2

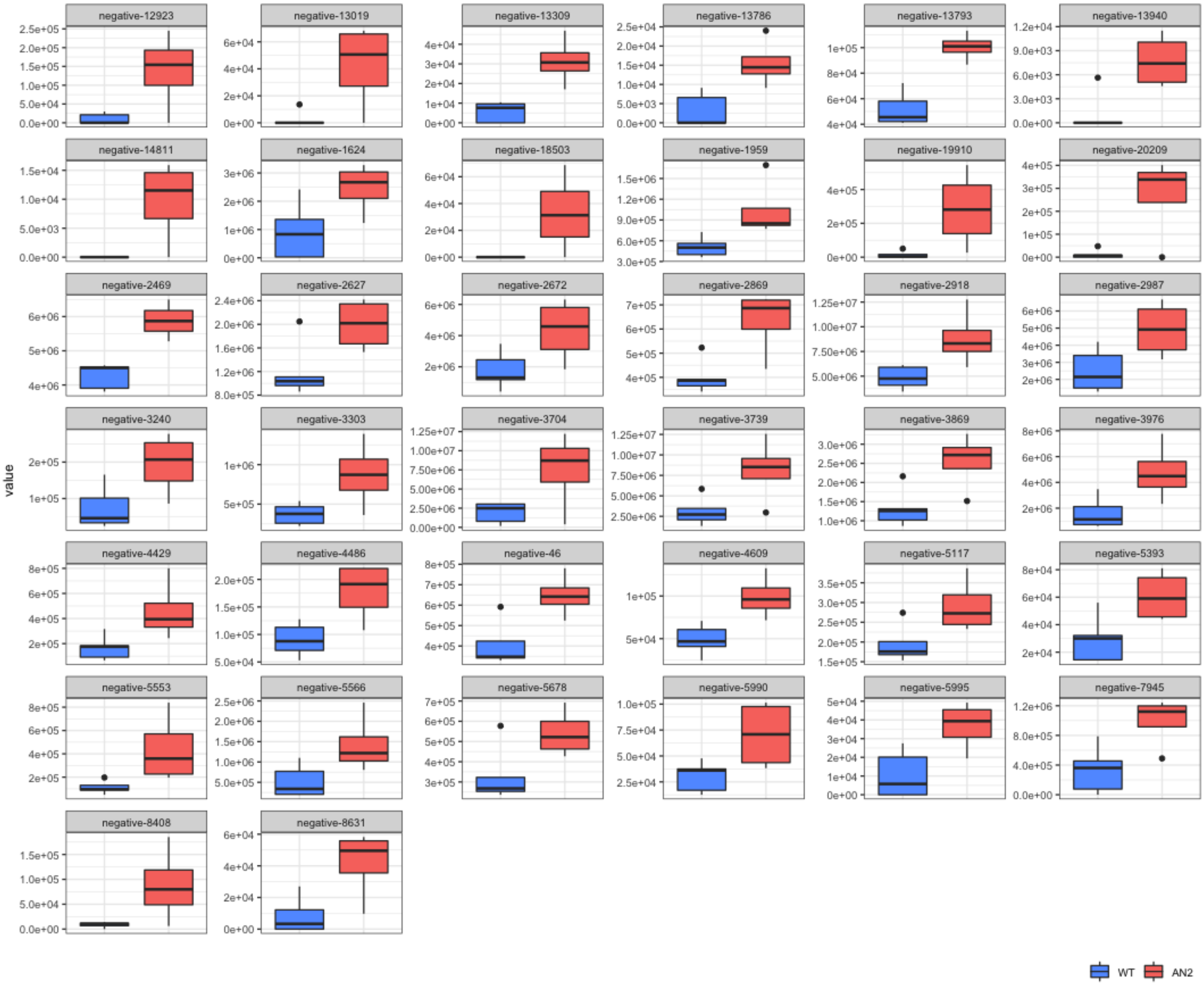

negative-12923
negative-13019
negative-13309
negative-13786
negative-13793
negative-13940
negative-14811
negative-1624
negative-18503
negative-1959
negative-19910
negative-20209
negative-2469
negative-2627
negative-2672
negative-2869
negative-2918
negative-2987
negative-3240
negative-3303
negative-3704
negative-3739
negative-3869
negative-3976
negative-4429
negative-4486
negative-46
negative-4609
negative-5117
negative-5393
negative-5553
negative-5566
negative-5678
negative-5990
negative-5995
negative-7945
negative-8408
negative-8631
value
WT
AN2

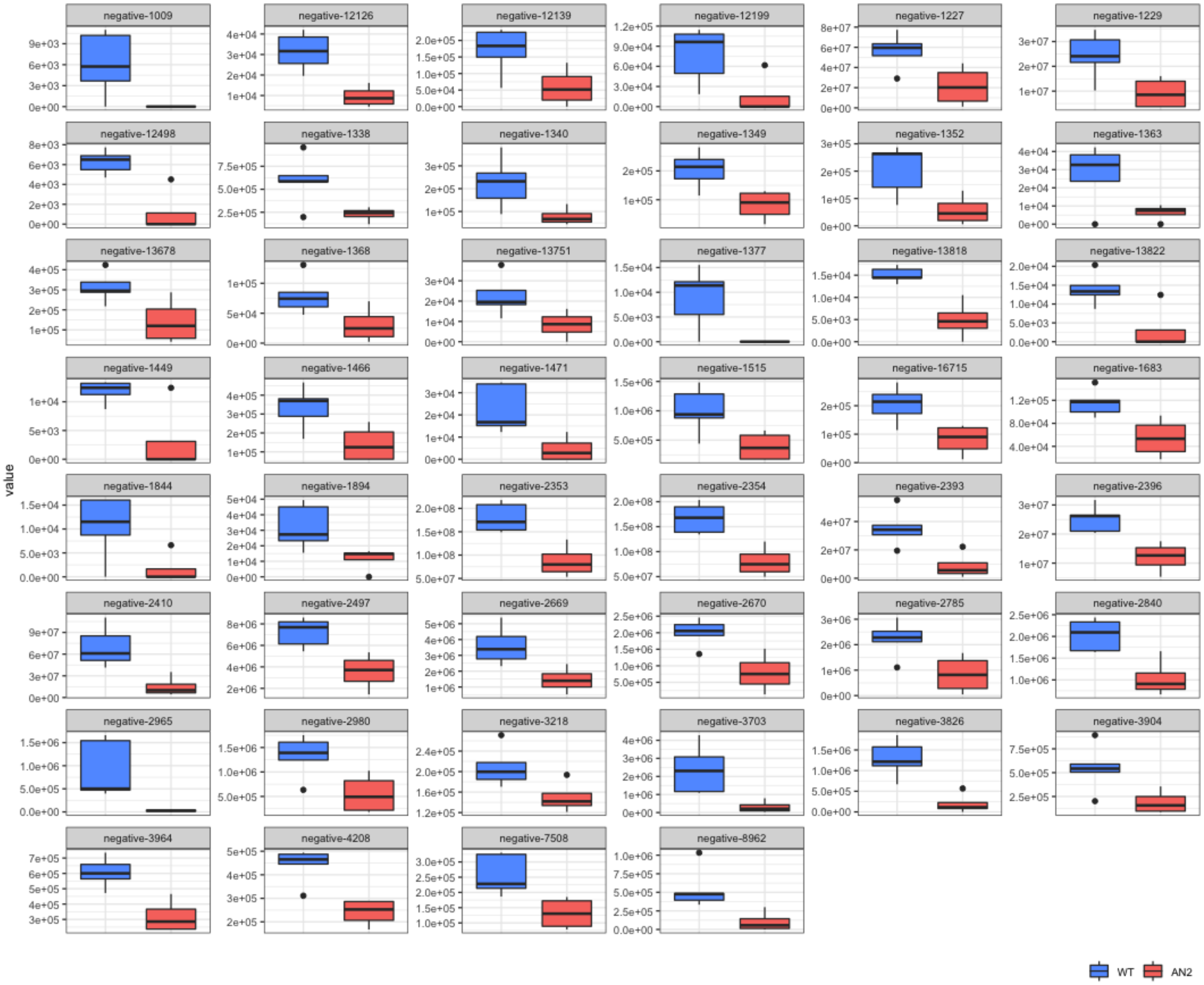

Supplemental Fig. 4: Feature intensity of features found to be enriched or depleted in WT vs. Zman2; n = 5 for WT, n = 4 for Zman2 in positive and negative ionization modes of LC/MS-MS.
